# Association of Maternal and Child Anemia With Brain Structure in Early Life in South Africa

**DOI:** 10.1001/jamanetworkopen.2022.44772

**Published:** 2022-12-02

**Authors:** Catherine J. Wedderburn, Jessica E. Ringshaw, Kirsten A. Donald, Shantanu H. Joshi, Sivenesi Subramoney, Jean-Paul Fouche, Jacob A. M. Stadler, Whitney Barnett, Andrea M. Rehman, Nadia Hoffman, Annerine Roos, Katherine L. Narr, Heather J. Zar, Dan J. Stein

**Affiliations:** 1 Department of Paediatrics and Child Health, Red Cross War Memorial Children’s Hospital, University of Cape Town, Cape Town, South Africa; 2 Neuroscience Institute, University of Cape Town, Cape Town, South Africa; 3 Department of Clinical Research, London School of Hygiene & Tropical Medicine, London, United Kingdom; 4 Departments of Neurology, Psychiatry and Biobehavioral Sciences, University of California, Los Angeles; 5 Department of Bioengineering, University of California, Los Angeles; 6 South African Medical Research Council (SAMRC), Unit on Child & Adolescent Health, University of Cape Town, Cape Town, South Africa; 7 MRC International Statistics & Epidemiology Group, London School of Hygiene & Tropical Medicine, London, United Kingdom; 8 Department of Psychiatry & Mental Health, University of Cape Town, Cape Town, South Africa; 9 SA MRC Unit on Risk and Resilience in Mental Disorders, Department of Psychiatry, Stellenbosch University, Stellenbosch, South Africa; 10 SA MRC Unit on Risk and Resilience in Mental Disorders, Department of Psychiatry & Neuroscience Institute, University of Cape Town, Cape Town, South Africa

## Abstract

**Importance:**

Anemia affects millions of pregnant women and their children worldwide, particularly in low- and middle-income countries. Although anemia in pregnancy is a well-described risk factor for cognitive development, the association with child brain structure is poorly understood.

**Objective:**

To explore the association of anemia during pregnancy and postnatal child anemia with brain structure in early life.

**Design, Setting, and Participants:**

This neuroimaging nested cohort study was embedded within the Drakenstein Child Health Study (DCHS), a population-based birth cohort in South Africa. Pregnant individuals were enrolled into the DCHS between 2012 and 2015 from 2 clinics in a periurban setting. Mother-child pairs were assessed prospectively; follow-up is ongoing. A subgroup of children had brain magnetic resonance imaging (MRI) at age 2 to 3 years from 2015 to 2018. This study focused on the 147 pairs with structural neuroimaging and available hemoglobin data. Data analyses were conducted in 2021 and 2022.

**Exposures:**

Mothers had hemoglobin measurements during pregnancy, and a subgroup of children had hemoglobin measurements during early life. Anemia was classified as hemoglobin levels less than 11 g/dL based on World Health Organization guidelines; children younger than 6 months were classified using local guidelines.

**Main Outcomes and Measures:**

Child brain volumes of global, subcortical, and corpus callosum structures were quantified using T1-weighted MRI. Linear regression models were used to analyze the associations between maternal and child anemia with child brain volumes, accounting for potential confounders.

**Results:**

Of 147 children (mean [SD] age at MRI, 34 [2] months; 83 [56.5%] male) with high-resolution MRI scans, prevalence of maternal anemia in pregnancy was 31.3% (46 of 147; median [IQR] gestation of measurement: 13 [9-20] weeks). Maternal anemia during pregnancy was significantly associated with smaller volumes of the child caudate bilaterally (adjusted percentage difference, −5.30% [95% CI, −7.01 to −3.59]), putamen (left hemisphere: −4.33% [95% CI, −5.74 to −2.92]), and corpus callosum (−7.75% [95% CI, −11.24 to −4.26]). Furthermore, antenatal maternal hemoglobin levels were also associated with brain volumes in the caudate (left hemisphere: standardized β = 0.15 [95% CI, 0.02 to 0.28]; right hemisphere: β = 0.15 [95% CI, 0.02 to 0.27]), putamen left hemisphere (β = 0.21 [95% CI, 0.07 to 0.35]), and corpus callosum (β = 0.24 [95% CI, 0.09 to 0.39]). Prevalence of child anemia was 52.5% (42 of 80; median [IQR] age of measurement: 8.0 [2.7 to 14.8] months). Child anemia was not associated with brain volumes, nor did it mediate the association of maternal anemia during pregnancy with brain volumes.

**Conclusions and Relevance:**

In this cohort study, anemia in pregnancy was associated with altered child brain structural development. Given the high prevalence of antenatal maternal anemia worldwide, these findings suggest that optimizing interventions during pregnancy may improve child brain outcomes.

## Key Points

**Question:**

Are antenatal maternal anemia and postnatal child anemia associated with brain volumes in early childhood?

**Findings:**

In this cohort study of 147 mother-child pairs in South Africa, there was a 31% prevalence of maternal anemia in pregnancy. Maternal anemia, but not child anemia, was associated with significantly smaller subcortical (caudate and putamen) and corpus callosum volumes in children at ages 2 to 3 years.

**Meaning:**

These findings suggest that anemia during pregnancy was associated with altered brain structure in children, highlighting the importance of optimizing interventions during pregnancy.

## Teaser

This cohort study investigates associations of anemia during pregnancy and postnatal child anemia with brain structure among children aged 2 to 3 years.

## Introduction

Anemia is a major health burden affecting approximately 273 million people.^1^ Women and children are particularly vulnerable.^2^ Globally, 38% of pregnant women and 43% of children are estimated to be anemic,^1^ with the highest prevalence in low- and middle-income countries (LMICs).^3,4,5,6^ Anemia is defined by low hemoglobin concentration, and the most common cause is iron deficiency, representing half of cases.^1,6^

Anemia in pregnancy is a well-described risk factor for poor maternal and infant health outcomes including increased risk of maternal mortality, stillbirth, prematurity, and low birthweight.^3,7,8^ Furthermore, it is recognized as a leading cause of lost developmental potential in LMICs.^2^ Findings from the South African Drakenstein Child Health Study (DCHS) suggest antenatal maternal anemia is a key driver of poor child neurocognitive outcomes,^9^ supporting global reports.^10,11^ Studies have also shown that child anemia may affect long-term academic achievement despite supplementation.^12,13^ However, little is known about the association of anemia with child brain structure, and the relative influence of antenatal compared with postnatal anemia. Although effective interventions for anemia exist,^14^ these could be optimized with further understanding of the impact on child brain development.^2,15^

Neuroimaging may provide insights into the association of anemia with early brain maturation and the neurobiology underpinning developmental outcomes.^16^ However, few magnetic resonance imaging (MRI) studies have investigated the association of anemia with developing brain outcomes and, of these, most have focused on child anemia in the postnatal period.^17,18,19^ Furthermore, to our knowledge, no studies have differentiated between the associations of anemia in pregnancy vs anemia in childhood. In neonates, one study in India reported an association between antenatal anemia and smaller hippocampal volumes,^20^ and a recent US study identified an association between maternal iron intake and cortical microstructure.^21^ To our knowledge, no studies have been conducted in sub-Saharan Africa, where loss of neurodevelopmental potential is greatest.^15^

The DCHS provides a unique opportunity to investigate the association of anemia with brain development during critical windows of vulnerability where interventions may have the most benefit. We aimed to examine the associations among maternal anemia during pregnancy, postnatal child anemia, and child brain structure at age 2 to 3 years in a subgroup of this South African birth cohort.

## Methods

This cohort study was approved by the University of Cape Town Human Research Ethics Committee, Stellenbosch University, and the Western Cape Provincial Health Research Committee. Written informed consent was obtained from all pregnant individuals at enrollment. The present study followed the Strengthening the Reporting of Observational Studies in Epidemiology (STROBE) reporting guideline.

### Design and Setting

The population-based DCHS birth cohort is located in a periurban district 60 km outside of Cape Town, South Africa.^22,23^ The community is characterized by low socioeconomic status, with high unemployment and multiple health and psychosocial risk factors, representative of many communities in South Africa and other LMICs. More than 90% of the population accesses public health services. Pregnant individuals aged at least 18 years were recruited while attending routine antenatal care at 2 public sector primary health care clinics, Mbekweni and TC Newman, which lie at sea level.

### Participants

The DCHS enrolled 1225 pregnant individuals between 2012 and 2015. A total of 1143 livebirths were included and are being followed postnatally with good retention in care.^24^ A study flowchart is included in the eFigure in Supplement 1. A subgroup of children participated in a nested longitudinal neuroimaging substudy embedded in the larger DCHS, with the first neuroimaging time point at 2 to 6 weeks of age. Of eligible children, those who underwent MRI in the neonatal period were included at 2 to 3 years of age. In addition, children not imaged at birth were also selected based on known risk factors for neurodevelopment in this population (maternal HIV^25^ and alcohol use in pregnancy^26^), and a randomly selected comparison group.^27^ A total of 239 children were invited to attend neuroimaging after turning 2 years of age (2015 to 2018),^27^ with exclusion criteria: (1) medical comorbidity (genetic syndrome, neurological disorder, or congenital abnormality); (2) gestation less than 36 weeks; (3) low Apgar score (less than 7 at 5 minutes); (4) neonatal intensive care admission; (5) maternal use of illicit drugs; (6) MRI contraindications; and (7) child HIV infection. The nested neuroimaging group was comparable with the full birth cohort (see Wedderburn et al^27^). T1-weighted MRI images were acquired on 162 children, of which 147 had maternal hemoglobin data and 80 had child hemoglobin data.

### Measures

Comprehensive biomedical, environmental, psychosocial, demographic, and physical data of the mother and child were collected antenatally. Birth information was abstracted by study staff at delivery. Child gestational age at delivery was calculated based on antenatal ultrasonography, if available; otherwise gestational age was determined using symphysis-fundal height or maternal report of last menstrual period. All mothers were tested for HIV during pregnancy with repeat testing along with infant testing during the postnatal period per national guidelines. Child anthropometry measurements were taken at routine study visits at 2 years according to World Health Organization (WHO) standards.^28^ Maternal weight and body mass index were measured at 6 weeks postpartum. Maternal smoking in pregnancy was assessed through self-report. A dichotomous classification of alcohol use was generated using the Alcohol, Smoking and Substance Involvement Screening Test and a retrospective questionnaire on alcohol use.

Mothers had hemoglobin levels measured during pregnancy when they attended their antenatal booking visit as standard-of-care, where iron (ferrous sulfate) and folic acid supplementation are given per national guidelines. Hemoglobin was measured using rapid tests, and levels were abstracted from antenatal clinical records by trained study staff at DCHS enrollment visit. Based on WHO guidelines,^29^ hemoglobin levels less than 11 g/dL (to convert to grams per liter, multiply by 10.0) during pregnancy were classified as anemia. Further classifications into mild (10.0 to 10.9 g/dL), moderate (7.0 to 9.9 g/dL), and severe anemia (less than 7.0 g/dL) were made.^29^ Children had hemoglobin measured as a laboratory full blood count if they presented to a hospital with pneumonia between birth and the MRI scan. For children with multiple measurements, the lowest hemoglobin measurement was used to reflect the most severe presentation. Child anemia was defined using WHO guidelines for all children aged older than 6 months.^29^ For children younger than 6 months, age-specific cutoffs for anemia were taken from local (Groote Schuur Hospital/University of Cape Town Pathology Laboratory) guidelines (eTable 2 in Supplement 1).

### Neuroimaging

Neuroimaging was conducted at the Cape Universities Body Imaging Centre using a 3-Tesla Siemens Skyra whole body MRI scanner (Siemens) and 32-channel head-coil. Children were scanned during natural sleep without sedation. The neuroimaging protocol and MRI specifications are detailed in the eAppendix in Supplement 1.^27^

Structural MRIs were processed using FreeSurfer version 6.0 software (Laboratory for Computational Neuroimaging at the Athinoula A. Martinos Center for Biomedical Imaging) using the automated processes of cortical reconstruction and volumetric segmentation.^30,31^ Subcortical and cortical tissue volumes were extracted for analysis. Based on previous research findings,^17,19,20,21^ we focused on global volumes (cerebral white matter, total gray matter, subcortical gray matter), subcortical structures (thalamus, caudate, putamen, pallidum, amygdala, hippocampus, nucleus accumbens), and the corpus callosum. The corpus callosum was segmented into posterior, midposterior, central, midanterior, and anterior regions,^30,31,32^ which were investigated independently. The midposterior, central, and midanterior volumes were summed to make the body, and the total corpus callosum volume was calculated by summing all anatomical regions.^33^ Intracranial volume was included in analyses to account for brain size.^34,35^

### Statistical Analysis

**Figure 1.**
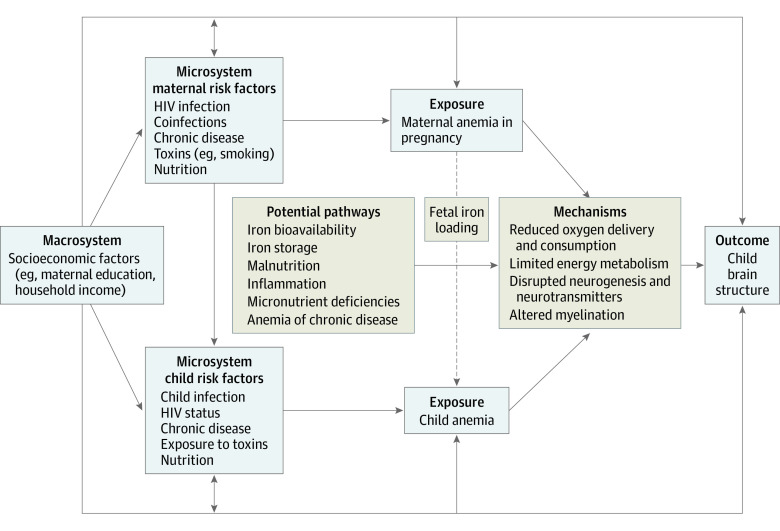
Conceptual Framework Model of Potential Pathways Connecting Maternal Anemia and Child Anemia With Child Brain Structure This model was developed using multiple information sources.^3,9,36,37,38,39,40,41^ We performed multivariable analyses adjusting for potential confounding factors at the macrosystem level, and sensitivity analyses including potential confounders at the microsystem level where available.

Sample characteristics and clinical variables are presented as means (with SDs) for continuous data and frequencies for categorical data. We compared sociodemographic characteristics between children born to mothers with anemia during pregnancy vs those whose mothers did not have anemia during pregnancy using unpaired *t* tests for continuous data and χ^2^ tests for categorical data. The primary exposure was maternal anemia during pregnancy (dichotomized by hemoglobin levels less than 11 g/dL vs 11 g/dL or higher; independent variable) and primary outcomes were child brain volumes (dependent variables). We first reported mean differences and Cohen *d* effect sizes by maternal anemia status using independent linear regression models for each region of interest. Models were adjusted for child age at MRI, child sex, intracranial volume, maternal education, and household income reported antenatally, ie, factors known to influence brain development selected a priori, as represented in our conceptual framework^3,9,36,37,38,39,40,41^ (Figure 1). In regions where an association was observed (*P* < .05), we calculated adjusted percentage differences and explored multivariable linear regression models using standardized regression coefficients for continuous maternal hemoglobin concentrations and, separately, categorical anemia severity.^29^ Additionally, we investigated child anemia as our exposure across all regions of interest and conducted mediation analyses using structural equation modeling. We checked normality of residuals and homogeneity of variance for each model using Q-Q plots and visualizing a scatterplot to ensure robustness of model assumptions.

To validate our analyses, we performed several sensitivity analyses. First, given that tobacco smoking is known to increase hemoglobin concentrations,^29^ standard anemia cutoffs may underestimate functional anemia in people who smoke; therefore, we adjusted hemoglobin concentrations of individuals who were active smokers in pregnancy per WHO guidelines. Second, in healthy individuals, given hemoglobin concentrations change over pregnancy trimesters as a result of higher maternal blood volume,^29^ we also adjusted for trimester of pregnancy of hemoglobin measurement. Third, to ensure robustness against unmeasured confounding, we built additional multivariable models adjusting for maternal alcohol use in pregnancy, given the known impact on child brain structure,^42^ and maternal HIV status, given high HIV prevalence in our setting.

All analyses were performed using Stata version 14.2 (StataCorp). A 2-sided significance level of *P* < .05 was used throughout; adjusted analyses are presented. Bonferroni corrections were reported for 14 subcortical regional models and 7 corpus callosum regional models. Data analyses were performed 2021 to 2022.

## Results

### Maternal Anemia and Child Brain Structure

A total of 147 children with MRI (mean [SD] child scan age 34 [2] months, 83 [56.5%] male) were born to mothers with hemoglobin measured during pregnancy. Maternal hemoglobin measurements were mainly taken in the first (50.3%) and second trimesters (46.9%) of pregnancy, at a median (IQR) of 13 (9-20) weeks’ gestation. Overall, 46 of 147 mothers (31.3%) were found to have anemia in pregnancy. Of these, 24 of 46 mothers (52.2%) had mild anemia, and 22 of 46 mothers (47.8%) had moderate anemia (Table 1). Sociodemographic characteristics, antenatal exposures including smoking and alcohol use in pregnancy, and maternal and child anthropometry were similar between groups (Table 1). HIV infection was prevalent in mothers with anemia (23 mothers [50.0%]) and without anemia (44 mothers [43.6%]); all mothers with HIV infection were receiving antiretroviral drug regimens (66 of 67 mothers [98.5%] receiving triple therapy).

**Table 1.** Sociodemographic Characteristics of Children Born to Mothers With Anemia in Pregnancy vs Without Anemia in Pregnancy^a^

Variable	No. (%)		*P* value
	Maternal anemia (n = 46)	No maternal anemia (n = 101)	
**Pregnancy characteristics**			
Anemia in pregnancy^b^			
Mild	24 (52.2)	NA	NA
Moderate	22 (47.8)	NA	NA
Severe	0	NA	NA
Hemoglobin during pregnancy, mean (SD), g/dL	9.8 (0.7)	12.3 (0.9)	<.001
Trimester of pregnancy measured^c^			
First	13 (28.3)	61 (60.4)	.001
Second	31 (67.4)	38 (37.6)	
Third	2 (4.4)	2 (2.0)	
**Maternal sociodemographic characteristics**			
Site (TC Newman)	11 (23.9)	24 (23.8)	.98
Monthly household income, ZAR			
<1000 (approximately <$75)	19 (41.3)	28 (27.7)	.10
>1000 (approximately >$75)	27 (58.7)	73 (72.3)	
Education			
Any secondary	34 (73.9)	65 (64.4)	.25
Completed secondary	12 (26.1)	36 (35.6)	
Employed	11 (23.9)	31 (30.7)	.40
Age at delivery, mean (SD), y	28.9 (6.5)	28.3 (5.2)	.55
Smoking during pregnancy	6 (13.0)	19 (18.8)	.39
Alcohol use during pregnancy	7 (19.4)	13 (15.9)	.63
HIV infection	23 (50.0)	44 (43.6)	.47
Weight 6 wk postpartum, mean (SD), kg	70.6 (20.2)	76.2 (16.1)	.14
BMI 6 wk postpartum, mean (SD)	28.2 (6.9)	29.6 (6.2)	.31
**Child characteristics** ^d^			
Age at scan, mean (SD), mo	33.8 (2.0)	34.2 (1.6)	.19
Sex			
Male	28 (60.9)	55 (54.5)	.47
Female	18 (39.1)	46 (45.5)	
HIV infection	0	0	NA
Gestational age at birth, mean (SD), wk	38.8 (2.8)	38.9 (2.5)	.84
Birth weight, mean (SD), kg	3.06 (0.57)	3.09 (0.59)	.79
Birth length, mean (SD), cm	49.3 (3.6)	49.5 (4.1)	.86
Birth head circumference, mean (SD), cm	33.6 (2.2)	33.6 (1.9)	.98
WAZ at 2 y, mean (SD)	0.15 (1.2)	−0.12 (1.3)	.27
Underweight at 2 y	2 (5.3)	6 (6.5)	.79
HAZ at 2 y, mean (SD)	−0.95 (1.2)	−0.88 (1.1)	.77
Stunting at 2 y	7 (18.4)	14 (15.2)	.65
HCZ at 2 y, mean (SD)	0.57 (1.6)	0.24 (1.5)	.26
Microcephaly at 2 y	2 (5.4)	4 (4.4)	.80
Total intracranial volume, mean (SD), cm^3^	1214 (128)	1211 (113)	.88

Abbreviations: BMI, body mass index (calculated as weight in kilograms divided by height in meters squared); HAZ, *z*-scores for height-for-age; HCZ, *z*-scores for head circumference-for-age ; WAZ, *z*-scores for weight-for-age; ZAR, South African Rand.

SI conversion factor: To convert hemoglobin grams per liter, multiply by 10.

^a^ Continuous variables were compared with unpaired *t* tests; categorical variables were compared with χ^2^ tests. Percentages are cited among those with nonmissing values. Data were missing for 29 women for maternal alcohol use; 45 women for maternal BMI and weight 6 weeks postpartum; 1 child for birth weight; 4 children for birth length; 3 children for birth head circumference; 17 children for WAZ and HAZ at age 2 years; and 18 children for HCZ at age 2 years.

^b^ Anemia was classified per the WHO recommendations as hemoglobin less than 11 g/dL. Subcategories were defined as mild, 10.0 to 10.9 g/dL; moderate, 7.0 to 9.9 g/dL; and severe, less than 7.0 g/dL.

^c^ Trimester of pregnancy defined as first, 0 to 12 weeks; second, 13 to 27 weeks; and third, 28 weeks onwards.

^d^ The birth anthropometrical measurements were conducted by trained labor ward staff, the infant’s length was measured in cm to the nearest completed 0.5 cm, and their weight was measured in kg. Child weight and length measurements were converted to *z*-scores for WAZ, HAZ, and HCZ. Weight and length measurements were converted to *z*-scores based on age and sex, using Anthro software.^28^ Children were classified as underweight, stunted, or having microcephaly if they had a weight, height, or head circumference *z*-score less than −2 SDs, respectively.

**Figure 2.**
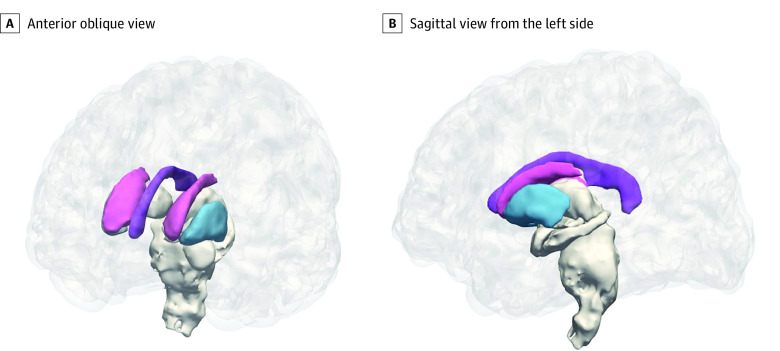
Subcortical and Corpus Callosum Volumes Associated With Maternal Anemia, Superimposed on a Cortical Surface Images were created using FreeSurfer. The colors represent the segmented structures as follows: purple, corpus callosum; pink, caudate nucleus; blue, putamen.

Maternal anemia in pregnancy was not associated with child global brain volumes after adjusting for covariates. However, we identified significant associations with individual subcortical structures, including smaller volumes of the child caudate bilaterally (left hemisphere adjusted percentage difference, −5.41% [95% CI, −7.21% to −3.60%]; Cohen *d*, −0.35 [95% CI, −0.70 to −0.00]; right hemisphere adjusted percentage difference, −5.20% [95% CI, −6.88% to −3.51%]; Cohen *d*, −0.34 [95% CI, −0.69 to 0.01]; overall adjusted percentage difference, −5.30% [95% CI, −7.01% to −3.59%]) and left putamen (adjusted percentage difference, −4.33% [95% CI, −5.74% to −2.92%]; Cohen *d*, −0.33 [95% CI, −0.68 to 0.02]) (Table 2 and Figure 2). Furthermore, we found an association between anemia in pregnancy and individual corpus callosum segments (central, anterior, midanterior, and posterior), with Cohen *d* effect sizes ranging from −0.36 to −0.40 (*P* for trend < .05). The association with the total corpus callosum volume (Cohen *d*, −0.44 [95% CI, −0.80 to −0.09]) remained significant after multiple comparison correction (Table 2 and Figure 2). The corpus callosum volume of children born to mothers with anemia in pregnancy was −7.75% (95% CI, −11.24% to −4.26%) smaller compared with the control group.

**Table 2.** Adjusted Mean Differences in Child Brain Volumes by Exposure to Maternal Anemia in Pregnancy

Brain volumes	Volume, mean (SD), mm^3^		Adjusted mean difference (95% CI)^a^	*P* value	Effect size, Cohen *d* (95% CI)
	No maternal anemia	Maternal anemia			
**Global volume**					
Cerebral white matter	310 884 (37 512)	310 824 (41 291)	−1543 (−8233 to 5147)	.65	−0.04 (−0.39 to 0.31)
Total gray matter	675 039 (55 008)	676 781 (61 973)	−766 (−9138 to 7606)	.86	−0.01 (−0.36 to 0.34)
Subcortical gray matter	47 930 (4205)	47 080 (4471)	−869 (−1804 to 66)	.07	−0.20 (−0.55 to 0.15)
**Subcortical regions**					
Thalamus					
Left	5922 (591)	5865 (585)	−48.89 (−189.65 to 91.87)	.49	−0.08 (−0.43 to 0.27)
Right	5830 (595)	5796 (542)	−21.25 (−165.81 to 123.30)	.77	−0.04 (−0.39 to 0.31)
Caudate					
Left	3302 (533)	3142 (407)	−178.82 (−316.42 to −41.22)	.01^d^	−0.35 (−0.70 to −0.00)
Right	3393 (542)	3241 (406)	−176.76 (−314.11 to −39.41)	.01^d^	−0.34 (−0.69 to 0.01)
Putamen					
Left	4524 (530)	4331 (689)	−195.95 (−380.76 to −11.14)	.04^d^	−0.33 (−0.68 to 0.02)
Right	4558 (528)	4450 (624)	−80.77 (−254.05 to 92.51)	.36	−0.14 (−0.49 to 0.21)
Pallidum					
Left	1604 (212)	1555 (271)	−57.67 (−129.06 to 13.71)	.11	−0.25 (−0.60 to 0.10)
Right	1529 (187)	1482 (234)	−48.48 (−109.99 to 13.03)	.12	−0.24 (−0.59 to 0.11)
Amygdala					
Left	1205 (149)	1192 (181)	−11.26 (−60.14 to 37.62)	.65	−0.07 (−0.42 to 0.28)
Right	1325 (175)	1334 (170)	9.87 (−44.07 to 63.81)	.72	0.06 (−0.29 to 0.41)
Hippocampus					
Left	3066 (301)	3053 (324)	−12.63 (−99.71 to 74.45)	.78	−0.04 (−0.39 to 0.31)
Right	3144 (324)	3184 (403)	47.21 (−56.89 to 151.30)	.37	0.14 (−0.21 to 0.48)
Accumbens					
Left	569 (95)	584 (105)	13.01 (−18.04 to 44.06)	.41	0.13 (−0.22 to 0.48)
Right	577 (94)	589 (87)	8.36 (−20.91 to 37.63)	.57	0.09 (−0.26 to 0.44)
Corpus callosum					
Posterior	639 (100)	597 (119)	−40.92 (−76.85 to −4.99)	.03^d^	−0.37 (−0.72 to −0.02)
Midposterior	332 (76)	317 (70)	−12.48 (−38.23 to 13.27)	.34	−0.17 (−0.52 to 0.18)
Central	428 (104)	393 (87)	−35.97 (−70.83 to −1.11)	.04^d^	−0.36 (−0.71 to −0.00)
Midanterior	422 (128)	379 (100)	−48.34 (−89.57 to −7.11)	.02^d^	−0.39 (−0.74 to −0.04)
Anterior	778 (163)	724 (142)	−64.02 (−114.22 to −13.82)	.01^d^	−0.40 (−0.75 to −0.05)
Body^b^	1182 (258)	1089 (223)	−96.79 (−182.23 to −11.35)	.03^d^	−0.38 (−0.73 to −0.03)
Total^c^	2600 (441)	2410 (430)	−201.73 (−345.55 to −57.92)	.006^d^^,^^e^	−0.44 (−0.80 to −0.09)

^a^ Multivariable linear regression was performed to assess the association of any maternal anemia with child brain volumes correcting for household income and maternal education, child age and sex, and intracranial volume. A negative mean difference represents smaller volumes in those children born to mothers with anemia.

^b^ Corpus callosum body: sum of midposterior, central, and midanterior regions.

^c^ Corpus callosum total: sum of posterior, midposterior, central, midanterior, and anterior regions.

^d^ *P* < .05 uncorrected.

^e^ *P* values survive Bonferroni correction (subcortical cutoff: *P* < .004; Corpus callosum: *P* < .007).

**Figure 3.**
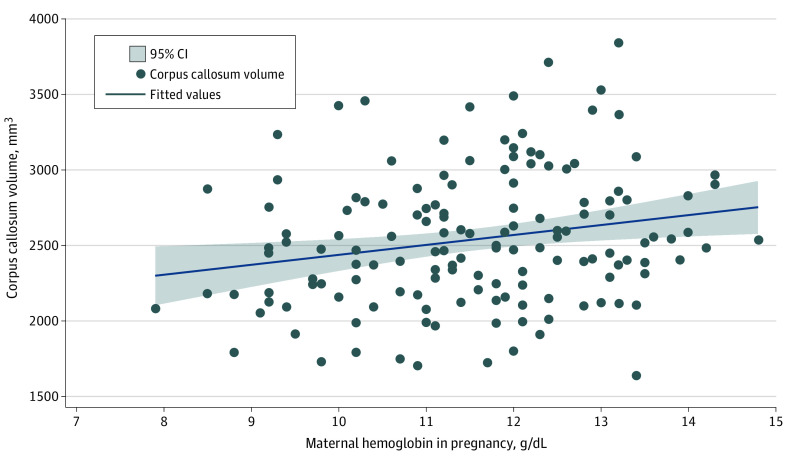
Linear Regression of Total Corpus Callosum Volume by Maternal Hemoglobin in Pregnancy Linear regression of maternal hemoglobin concentration (in grams per deciliter; to convert to grams per liter, multiply by 10) in pregnancy with child total corpus callosum volume (in millimeters cubed) with line of best fit and 95% CIs, unadjusted.

Similarly, lower maternal hemoglobin levels were associated with smaller caudate volume (left hemisphere: β = 0.15 [95% CI, 0.02 to 0.28]; right hemisphere: β = 0.15 [95% CI, 0.02 to 0.27]), left hemisphere putamen volume (β = 0.21 [95% CI, 0.07 to 0.35]) and corpus callosum total volume (β = 0.24 [95% CI, 0.09 to 0.39]) (Figure 3) on multiple regression accounting for covariates. Furthermore, children born to mothers with moderate anemia had significantly lower regional volumes than those with mild anemia (eTable 1 in Supplement 1).

### Child Anemia and Brain Structure

Child hemoglobin levels were available on a subgroup of 80 children with imaging (median [IQR] age at hemoglobin measurement: 8.0 [2.7-14.8] months). Of these children, 42 of 80 (52.5%) were classified as anemic (eTable 2 in Supplement 1). Sociodemographic characteristics were similar between children with and without anemia, as well as between the subgroup of children with hemoglobin measurements and those without (eTables 3 and 4 in Supplement 1). There were no statistically significant associations identified between child anemia alone and global, subcortical, or corpus callosum volumes in this cohort (eTable 5 in Supplement 1).

### Maternal and Child Anemia and Child Brain Structure

To understand whether child anemia mediates the association between maternal anemia and child brain structure, we used structural equation modeling. There was no association identified between maternal anemia in pregnancy and child anemia (χ^2^ = 0.004, *P* = .95) (eTable 6 in Supplement 1), and no evidence for mediation (eTable 7 in Supplement 1).

### Sensitivity Analyses

The identified associations between maternal anemia and subcortical and corpus callosum volumes were found to be robust in sensitivity analyses. The coefficients held after adjusting maternal hemoglobin concentration for smoking and adjusting for alcohol use in pregnancy, maternal HIV status, and trimester of pregnancy at hemoglobin measurement in the respective analyses.

## Discussion

In this neuroimaging nested cohort study of mother-child pairs in South Africa, 31% of mothers were found to have anemia in pregnancy (hemoglobin less than 11 g/dL). At age 2 to 3 years, children born to mothers with antenatal anemia had smaller basal ganglia (caudate and putamen) and corpus callosum volumes compared with children of mothers without anemia. Furthermore, maternal antenatal hemoglobin levels were significantly associated with child brain volumes in these regions. In contrast, postnatal child anemia was not found to be associated with subcortical or cortical brain volumes examined in this cohort. Overall, this suggests that antenatal maternal anemia, even when mild, was associated with altered child brain development.

Although maternal anemia has been associated with poor child neurocognitive outcomes in multiple settings,^9,10,11^ there are limited studies exploring the role of anemia in child brain structural development. We found that antenatal maternal anemia was associated with smaller volumes in deep gray matter structures (basal ganglia nuclei) and a major white matter structure (corpus callosum) in children aged 2 to 3 years. The marked magnitude of effect size, particularly in the corpus callosum, is important, given that the association was observed even though no mothers in the group had severe anemia. Previous studies of maternal anemia and brain development have not examined the basal ganglia or corpus callosum.^20^ However, changes in the corpus callosum correspond with research identifying an association between severe iron-deficient anemia in infants and a white matter predominant pattern of injury in the brain.^17^ In this cohort we found no evidence of mediation between maternal and child anemia, or associations between child anemia and brain volumes. Our findings suggest that there may be specific brain regions most susceptible to anemia and that the timing of exposure is critical, with in utero emerging as the most sensitive period in our study.

There is particular functional significance associated with volumetric changes in the basal ganglia and corpus callosum given that these structures are known to contribute to multiple neurocognitive domains including motor skills, executive functioning, and visuospatial ability.^43,44,45^ The corpus callosum is the largest myelinated bundle in the brain, connecting the 2 cerebral hemispheres, and playing a critical role in transmitting information.^46^ Studies have shown alterations in this brain region are associated with deficits in key cognitive functions.^47,48^ Furthermore, associations between iron-rich basal ganglia structures and spatial intelligence in school-age children have been identified,^19^ providing a potential neurobiological mechanism for reported visuospatial deficits in iron-deficient infants^49,50^ and adults.^51^

The mechanisms by which anemia may affect child brain development are complex and potentially overlapping. Anemia may reduce the hemoglobin-facilitated delivery and consumption of oxygen in the developing brain, particularly during pregnancy when there is an expanded blood volume and increased metabolic demand. There are multiple underlying etiologies of anemia, including micronutrient deficiencies (eg, iron, B_12_), infections (eg, HIV, tuberculosis), and inflammation, each with separate mechanisms that may additionally affect brain development.^3,37^ The most common of these is iron deficiency anemia, which can be due to a total reduction in iron stores or insufficient bioavailability of iron.^38,39,52^ This is worsened in individuals who have iron deficiency prior to pregnancy^53^ and in the context of HIV infection, for whom chronic inflammation may increase iron sequestration.^38,39,54,55^ Iron plays an important biological role in neurogenesis,^10,37^ neurotransmitter systems,^56^ gene expression and regulation,^57,58,59^ myelination,^60^ and energy metabolism.^37^ Deficiency is known to disrupt the dopaminergic neurotransmitter system of the basal ganglia while simultaneously reducing global myelination of the white matter,^40^ consistent with our results.

Anemia is generally treatable using simple interventions, such as iron supplementation, which increase hemoglobin concentration over time.^61^ However, the timing of interventions aimed at improving child outcomes linked to anemia has been controversial.^62^ Studies have suggested that child postnatal iron supplementation does not improve cognitive development in children aged 2 to 3 years living in LMICs^13^ or necessarily have long-term cognitive benefits, as shown in reports of children followed up at ages 7 to 9 years in Thailand and Nepal.^12,63^ This supports our findings that childhood anemia may not be the driver in this association with neurodevelopmental outcomes, and that antenatal interventions may be key for ensuring optimal neurodevelopment. Therefore, our study reinforces the rationale for WHO recommendations of iron and folic acid in pregnancy.^14^ However, current supplementation dosage recommendations may not be sufficient for individuals presenting with anemia in pregnancy, or may be given too late, as many pregnant individuals may begin prenatal care beyond the first trimester. In recognizing the fetal origins of brain health, future research should explore optimizing antenatal interventions for lifelong impact, particularly where anemia is already present. Furthermore, additional strategies may be needed to combat the various determinants of anemia, including HIV infection. Given that anemia affects approximately one-third of pregnant women worldwide,^6^ and is a leading cause of lost developmental potential in LMICs,^2^ the imperative for effective, well-timed interventions is profound and should remain an international public health priority.

To our knowledge, this is the first study to differentiate the associations of maternal and child anemia with brain development. The data were obtained from a group of children with comprehensively characterized mother-child pairs followed longitudinally and presenting many methodological strengths. We examined both dichotomous anemia variables and continuous hemoglobin concentrations. We performed several sensitivity analyses adjusting hemoglobin concentrations to avoid underestimation of anemia in situations where hemoglobin concentrations may be raised.

### Limitations

Our study has some limitations. First, this subgroup represents a high-risk subset for anemia with enrichment for maternal HIV and alcohol use.^24^ However, these risk factors remain common in LMIC populations and therefore should not affect generalizability. Second, although this study cannot determine causality, we demonstrate robust sensitivity analyses, temporal associations, and biological plausibility using continuous hemoglobin values. Additionally, our comparisons of mild and moderate anemia are suggestive of a biological gradient. Hemoglobin levels during pregnancy were measured using rapid tests, which represents standard practice and, using point-of-care results, we were able to show an association. Third, the sample size for child anemia was small with lower variability which may have decreased power. In addition, this group represents children with pneumonia, which could have introduced selection bias; therefore, we cannot extrapolate these rates of anemia more broadly. Additionally, we acknowledge that there may be residual bias from other unmeasured factors. Longitudinal studies are needed to replicate these findings in different populations.

## Conclusions

The findings of this neuroimaging nested cohort study support the global recognition of anemia in pregnancy as a health priority. Maternal anemia, but not child anemia, was associated with smaller basal ganglia volumes and reduced corpus callosum size in children aged 2 to 3 years. Therefore, antenatal maternal anemia, even when in the mild range, may have persistent consequences for the developing brain. This finding emphasizes the fetal origins of brain health, highlighting the need to implement effective interventions targeting prevention and treatment of maternal anemia for improved child outcomes.

## Notes

Supplement 1. **eAppendix.** Neuroimaging Protocol and MRI Sequence Specification **eTable 1.** Child Brain Volumes Stratified by Maternal Anemia Severity **eTable 2.** Classification of Child Anemia by Age **eTable 3.** Sociodemographic Characteristics of Children With and Without Anemia **eTable 4.** Sociodemographic Characteristics of Children With and Without Hemoglobin Measurements **eTable 5.** Adjusted Mean Differences in Brain Volumes According to Child Anemia **eTable 6.** Number of Children With Both Maternal Antenatal and Child Hemoglobin Measurements **eTable 7.** Structural Equation Models **eFigure 1.** Drakenstein Child Health Study Flowchart

Supplement 2.
Data Sharing Statement
