# Supplementary material for: Association of Maternal and Child Anemia With Brain Structure in Early Life in South Africa
Source: JAMA Netw Open. 2022 Dec 2;5(12):e2244772. doi: 10.1001/jamanetworkopen.2022.44772 (PMC9719049; doi:10.1001/jamanetworkopen.2022.44772)
Supplement: Supplement 1. — eAppendix. Neuroimaging Protocol and MRI Sequence Specification eTable 1. Child Brain Volumes Stratified by Maternal Anemia Severity eTable 2. Classification of Child Anemia by Age eTable 3. Sociodemographic Characteristics of Children With and Without Anemia eTable 4. Sociodemographic Characteristics of Children With and Without Hemoglobin Measurements eTable 5. Adjusted Mean Differences in Brain Volumes According to Child Anemia eTable 6. Number of Children With Both Maternal Antenatal and Child Hemoglobin Measurements eTable 7. Structural Equation Models eFigure 1. Drakenstein Child Health Study Flowchart [file jamanetwopen-e2244772-s001.pdf]

Supplemental Online Content

Wedderburn CJ, Ringshaw JE, Donald KA, et al. Association of maternal and child anemia with brain structure in early life in South Africa. *JAMA Netw Open.* 2022;5(12):e2244772. doi:10.1001/jamanetworkopen.2022.44772

eAppendix. Neuroimaging Protocol and MRI Sequence Specification

eTable 1. Child Brain Volumes Stratified by Maternal Anemia Severity

eTable 2. Classification of Child Anemia by Age

eTable 3. Sociodemographic Characteristics of Children With and Without Anemia

eTable 4. Sociodemographic Characteristics of Children With and Without Hemoglobin Measurements

eTable 5. Adjusted Mean Differences in Brain Volumes According to Child Anemia

eTable 6. Number of Children With Both Maternal Antenatal and Child Hemoglobin Measurements

eTable 7. Structural Equation Models

eFigure 1. Drakenstein Child Health Study Flowchart

This supplemental material has been provided by the authors to give readers additional information about their work.

© 2022 Wedderburn CJ et al. *JAMA Network Open.*

eAppendix. Neuroimaging protocol and MRI sequence specification

Neuroimaging

3D T1-weighted Multi-Echo Magnetization Prepared Rapid Acquisition Gradient Echo (MEMPRAGE) images were collected in sagittal orientation with the following parameters: repetition time=2530ms; echo time=1.69, 3.54, 5.39, 7.24ms; flip angle=7.0°; voxel size 1.0x1.0x1.0mm^3; inversion time=1100ms; field of view=224x224x176mm; 176 slices, 1.0mm thick. Scan time: 5min21s.

Quality control was performed and T1 images were checked for movement, along with FreeSurfer outputs for segmentation errors. A radiologist reviewed child scans for qualitative abnormalities and incidental findings were discussed with a paediatric neurologist and referred via established clinical pathways where relevant. These scans were excluded from analysis. Statistical quality checks were carried out using the ENIGMA pipeline (http://enigma.ini.usc.edu/protocols/imaging-protocols/).

© 2022 Wedderburn CJ et al. *JAMA Network Open.*

eTable 1: Child brain volumes stratified by maternal anemia severity

Brain volumes	Mean (SD) volume (mm^3)			
	No anemia	Mild anemia	Moderate anemia	Wald *p*-value [a]
Subcortical regions				
Caudate (bilateral) [b]	3348 (532)	3239 (449)	3139 (345)	0.04 [e]
Left	3302 (533)	3180 (472)	3101 (328)	0.04 [e]
Right	3393 (542)	3299 (432)	3177 (374)	0.04 [e]
Putamen (bilateral) [b]	4541 (502)	4544 (707)	4224 (521)	0.04 [e]
Left	4524 (530)	4495 (749)	4153 (583)	0.02 [e]
Right	4558 (528)	4593 (709)	4295 (486)	0.11
Corpus callosum				
Body [c]	1182 (258)	1149 (248)	1024 (175)	0.02 [e]
Total [d]	2600 (441)	2478 (472)	2336 (375)	0.02 [e]

Footnote: Mean volumes of child brain structures at 2-3 years stratified by severity of maternal anemia in pregnancy (n=147). Based on WHO guidelines, hemoglobin levels of <11g/dL during pregnancy classified women as anaemic. Further classifications into mild (hemoglobin 10.0 – 10.9g/dL) and moderate (hemoglobin 7.0 – 9.9g/dL) were made. No woman had severe anemia (hemoglobin <7.0g/dL).
[a] Adjusted for child age and sex, intracranial volume, maternal education and household income.[b] Mean of left and right hemispheres.
[c] Corpus callosum body: sum of mid-posterior, central and mid-anterior regions. [d] Corpus callosum total: sum of posterior, mid-posterior, central, mid-anterior and anterior regions. [e] $p<0.05$.

© 2022 Wedderburn CJ et al. *JAMA Network Open.*

eTable 2: Classification of child anemia by age

Child age at hemoglobin measurement (hemoglobin concentration cut-off for anemia)	Child anemia (n=42) No. (%)	No child anemia (n=38) No. (%)
0 – 3 days (<14g/dL)	1 (33.3)	2 (66.6)
3 days – 1 month (<15g/dL)	NA	NA
1 – 2 months (<11.5g/dL)	6 (75.0)	2 (25)
2 - 3 months (<9.4g/dL)	4 (33.3)	8 (66.6)
3 months – 2 years (<11.1g/dL)	30 (60.0)	20 (40)
2 – 6 years (<11g/dL)	1 (14.3)	6 (75)

Footnote: Reference Ranges were obtained from GSH/UCT Pathology Laboratory guidelines, Groote Schuur Hospital, National Health Laboratory Service (Western Cape); effective date 23 January 2003. WHO guidelines start from 6 months and classify children from 6 – 59 months with anemia if <11.0 g/dL. There were no differences when classifying children from 6 months using WHO guidelines.

© 2022 Wedderburn CJ et al. *JAMA Network Open.*

eTable 3: Sociodemographic characteristics of children with and without anemia

Variable	Child anemia (n=42) No. (%)	No child anemia (n=38) No. (%)	P-value
Sociodemographic characteristics			
Child age at scan, months, Mean (SD)	34.2 (1.9)	34.5 (1.4)	0.50
Sex (male)	20 (47.6)	25 (65.8)	0.10
Site (TC Newman)	9 (21.4)	13 (34.2)	0.20
Monthly household income (ZAR)			
< R1000 (<~$75)	13 (31.0)	11 (29.0)	0.85
>R1000 (>~$375)	29 (69.1)	27 (71.1)	
Maternal education			
Any Secondary	27 (64.3)	26 (68.4)	0.70
Completed secondary	15 (35.7)	12 (31.6)	
Maternal employment status (employed)	14 (33.3)	9 (23.7)	0.34
Maternal age at birth, years, mean (SD)	28.4 (5.6)	28.5 (4.8)	0.94
Gestational age at birth, weeks, mean (SD)	38.8 (2.6)	39.2 (2.7)	0.46
Birthweight, kg, mean (SD)	3.09 (0.67)	3.02 (0.65)	0.65
Birth length, mean (SD)	50.3 (3.3)	48.9 (4.0)	0.10
Birth head circumference, mean (SD)	33.9 (2.2)	33.3 (2.5)	0.31
WAZ at 2 years, mean (SD)	0.25 (1.4)	-0.23 (1.1)	0.10
Underweight at 2 years (<-2 z-scores)	2 (5.1)	1 (2.9)	0.64
HAZ at 2 years, mean (SD)	-0.89 (1.3)	-0.82 (1.1)	0.80
Stunting at 2 years (<-2 z-scores)	7 (18.0)	7 (20.6)	0.78
HCZ at 2 years, mean (SD)	0.64 (1.6)	0.36 (1.4)	0.44
Microcephaly at 2 years (<-2 z-scores)	2 (5.3)	1 (2.9)	0.62
Maternal smoking during pregnancy	6 (14.3)	8 (21.1)	0.43
Maternal alcohol use during pregnancy	5 (15.2)	9 (30.0)	0.16
Maternal HIV infection	20 (47.6)	13 (34.2)	0.22
Neuroanatomical variables			
Total Intracranial Volume (mm^3), Mean (SD)	1,231,536 (117,119)	1,210,409 (137,079)	0.46

Footnote: Data are n/N(%) or mean (SD). Continuous variables were compared with unpaired t-tests; categorical variables were compared with Chi-squared tests. Percentages calculated out of available data. Missing data: birthweight and head circumference (n=1); birth length (n=3); child WAZ and HAZ at 2 years (n=7); child HCZ at 2 years (n=8); maternal alcohol use in pregnancy (n=17). Child weight and length measurements were converted to z-scores for weight-for-age (WAZ), height-for-age (HAZ), and head circumference-for-age (HCZ). A cut-off of <-2 z-scores for these indices was used for classifying underweight, stunting or microcephaly.

© 2022 Wedderburn CJ et al. *JAMA Network Open.*

eTable 4: Sociodemographic characteristics of children with and without hemoglobin measurements

Variables	Neuroimaging & child hemoglobin measurements (n=80) No. (%)	Neuroimaging & no child hemoglobin measurements (n=82) No. (%)	*P*
Male sex	45 (56.3)	49 (59.8)	0.65
Site (TC Newman)	22 (27.5)	26 (31.7)	0.56
Monthly household income (ZAR)			
< R1000 (<~$75)	24 (30.0)	27 (32.9)	0.69
>R1000 (>~$75)	56 (70.0)	55 (67.1)	
Maternal education			
Secondary	53 (66.3)	55 (67.1)	0.91
Completed secondary	27 (33.8)	27 (32.9)	
Maternal employment status (employed)	23 (28.8)	21 (25.6)	0.65
Gestational age at birth, weeks, mean (SD)	39.0 (2.7)	38.7 (2.4)	0.54
Birthweight, kg, mean (SD)	3.06 (0.66)	3.12 (0.48)	0. 47
Birth length, cm, mean (SD)	49.6 (3.7)	49.4 (4.0)	0.74
Birth head circumference, cm, mean (SD)	33.6 (2.4)	33.7 (1.5)	0.81
WAZ at 2 years, mean (SD)	0.03 (1.2)	-0.21 (1.2)	0.24
Underweight at 2 years (<-2 z-scores)	3 (4.1)	5 (7.0)	0.44
HAZ at 2 years, mean (SD)	-0.86 (1.2)	-0.97 (1.06)	0.54
Stunting at 2 years (<-2 z-scores)	14 (19.2)	9 (12.7)	0.29
HCZ at 2 years, mean (SD)	0.51 (1.5)	0.15 (1.5)	0.16
Microcephaly at 2 years (<-2 z-scores)	3 (4.2)	4 (5.7)	0.67
Maternal smoking during pregnancy	14 (17.5)	18 (22.0)	0.48
Maternal alcohol use during pregnancy	14 (22.2)	10 (14.5)	0.25
Maternal HIV infection	33 (41.3)	37 (45.1)	0.62

Footnote: Data are n/N(%) or mean (SD). Continuous variables were compared with unpaired t-tests; categorical variables were compared with Chi-squared tests. Percentages were calculated out of available data. Missing data: birthweight (n=1); birth length (n=4); birth head circumference (n=3); child WAZ and HAZ (n=18); child HCZ (n=20); maternal alcohol use in pregnancy (n=30). Similar results were seen when comparing those children with both maternal and child hemoglobin measurements (n=75) and those without both measurements (87) with no significant differences between groups. Child weight and length measurements were converted to z-scores for weight-for-age (WAZ), height-for-age (HAZ), and head circumference-for-age. (HCZ) A cut-off of -2 z-scores for these indices was used for classifying underweight, stunting or microcephaly.

© 2022 Wedderburn CJ et al. *JAMA Network Open.*

eTable 5: Adjusted mean differences in brain volumes according to child anemia

Brain volumes		Adjusted [a] coefficient (95% CI)	*P*-value	Effect size
	Hemisphere			Cohen's *d* (95% CI)
Global volume				
Cerebral white matter	NA	2273 (-5826 to 10,374)	0.58	0.06 (-0.38 to 0.50)
Total grey matter	NA	798 (-10,088 to 11,684)	0.88	0.01 (-0.43 to 0.45)
Subcortical grey matter	NA	371 (-861 to 1604)	0.55	0.08 (-0.36 to 0.52)
Subcortical regions				
Thalamus	L	34.47 (-151.92 to 220.87)	0.71	0.05 (-0.38 to 0.49)
Thalamus	R	-16.31 (-218.24 to 185.62)	0.87	-0.03 (-0.46 to 0.41)
Caudate	L	-18.28 (-191.74 to 155.17)	0.83	-0.04 (-0.48 to 0.40)
Caudate	R	-77.75 (-249.87 to 94.36)	0.37	-0.16 (-0.60 to 0.28)
Putamen	L	-45.66 (-309.90 to 218.59)	0.73	-0.07 (-0.51 to 0.37)
Putamen	R	80.93 (-129.20 to 291.06)	0.45	0.13 (-0.31 to 0.57)
Pallidum	L	20.95 (-74.65 to 116.55)	0.66	0.08 (-0.36 to 0.52)
Pallidum	R	-18.36 (-99.35 to 62.63)	0.65	-0.08 (-0.52 to 0.36)
Amygdala	L	37.67 (-33.08 to 108.43)	0.29	0.21 (-0.23 to 0.65)
Amygdala	R	59.56 (-3.67 to 122.78)	0.06	0.35 (-0.10 to 0.79)
Hippocampus	L	70.57 (-63.21 to 204.35)	0.30	0.19 (-0.25 to 0.63)
Hippocampus	R	74.77 (-66.39 to 215.93)	0.30	0.19 (-0.25 to 0.63)
Accumbens	L	3.30 (-37.63 to 44.22)	0.87	0.03 (-0.41 to 0.47)
Accumbens	R	4.99 (-32.69 to 42.66)	0.79	0.05 (-0.39 to 0.49)
Corpus callosum				
Corpus callosum segments	Posterior	-12.86 (-67.70 to 41.97)	0.64	-0.10 (-0.54 to 0.33)
	Mid-Posterior	-6.29 (-39.53 to 26.96)	0.71	-0.09 (-0.53 to 0.35)
	Central	15.08 (-26.62 to 56.77)	0.47	0.17 (-0.27 to 0.61)
	Mid-Anterior	-12.62 (-68.16 to 42.92)	0.65	-0.10 (-0.54 to 0.34)
	Anterior	-51.13 (-111.58 to 9.32)	0.10	-0.34 (-0.78 to 0.10)
Body[b]		-3.83 (-111.27 to 103.61)	0.94	-0.02 (-0.45 to 0.42)
Total[c]		-67.82 (-260.78 to 125.14)	0.49	-0.15 (-0.59 to 0.29)

Footnote: Adjusted mean differences, *p*-values and Cohen's d effect sizes for the effects of child anemia on child brain structures (n=80). Brain volumes in (mm^3). [a] Multivariable linear regression was performed to assess the impact of child anemia on brain volumes correcting for child age and sex, ICV, household income and maternal education. [b] Corpus callosum body: sum of mid-posterior, central and mid-anterior regions; [c] Corpus callosum total: sum of posterior, mid-posterior, central, mid-anterior and anterior regions.

© 2022 Wedderburn CJ et al. *JAMA Network Open.*

eTable 6: Number of children with both maternal antenatal and child postnatal hemoglobin measurements

	Child Anemia		
**Maternal Anemia**	**Yes No. (%)**	**No No. (%)**	**Total**
**Yes**	14 (53.9)	12 (46.2)	26
**No**	26 (53.1)	23 (46.9)	49
**Total**	**40**	**35**	**75**

Footnote: Chi-squared test for the association between maternal and child anemia, p=0.95

© 2022 Wedderburn CJ et al. *JAMA Network Open.*

eTable 7: Structural equation models

Sobel method for testing the significance of the indirect mediation effect				
**Regions-of-interest**	**Indirect effect**	**Std. Err.**	**z-value**	**p-value**
Left caudate	-0.001 (95% CI -0.013, 0.011)	0.006	-0.159	0.874
Right caudate	-0.002 (95% CI -0.026, 0.022)	0.012	-0.163	0.871
Left putamen	-0.001 (95% CI -0.009, 0.007)	0.004	-0.147	0.883
Corpus callosum	-0.002 (95% CI -0.023, 0.019)	0.011	-0.162	0.871

Footnote. Significance testing of indirect effect (adjusted, standardized) using the Sobel method for each region that had a significant association with maternal anemia. No mediation was seen with any of the models. Similar results were obtained using the Monte Carlo test.

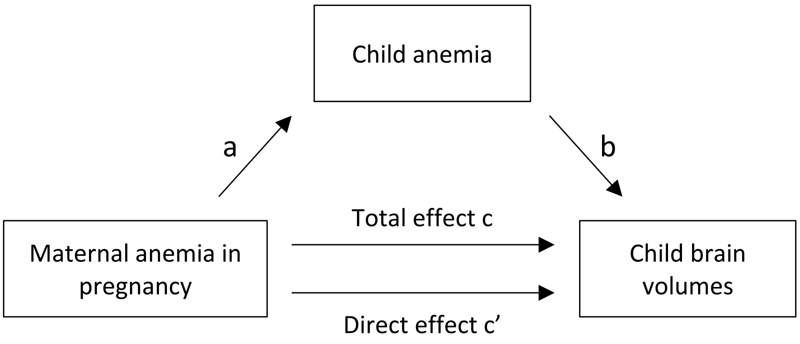

Illustration of mediation paths: Estimates of the total (path *c*), direct (path *c'*) and indirect (path *ab*; mediated through the influence on child anemia) effects of maternal anemia on child brain volume.

© 2022 Wedderburn CJ et al. *JAMA Network Open.*

eFigure 1. Drakenstein Child Health Study Flowchart

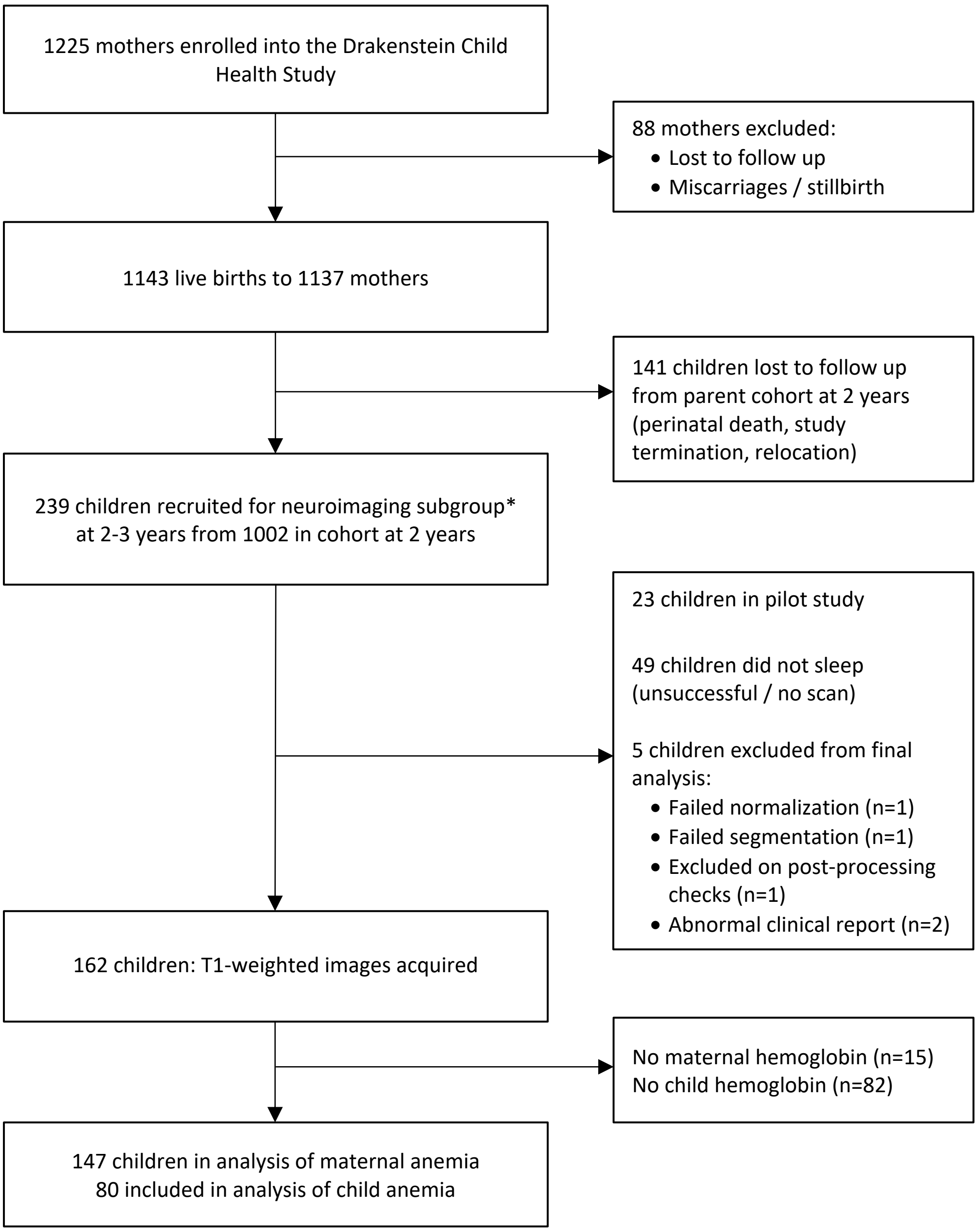

Footnote: Nested neuroimaging sub-study. Inclusion criteria: (i) currently active in the cohort, (ii) staying in the study area. Exclusion criteria: (i) Medical comorbidity (genetic syndrome, neurological disorder, or congenital abnormality); (ii) Gestation <36 weeks; (iii) Low Apgar score (<7 at 5 minutes); (iv) Neonatal intensive care admission; (v) Maternal use of illicit drugs during pregnancy; (vi) Child HIV infection. Selection criteria for children with neuroimaging at 2-3 years: Children with neonatal MRI were prioritised; children not imaged at birth were also selected based on risk factor exposure (maternal HIV and alcohol use during pregnancy) to ensure adequate representation, and a randomly selected comparison group frequency matched by age and sex.

© 2022 Wedderburn CJ et al. *JAMA Network Open.*
