# Supplementary material for: Association of Maternal and Child Anemia With Brain Structure in Early Life in South Africa
Source: JAMA Netw Open. 2022 Dec 2;5(12):e2244772. doi: 10.1001/jamanetworkopen.2022.44772 (PMC9719049; doi:10.1001/jamanetworkopen.2022.44772)
Supplement: Supplement 2. — Data Sharing Statement [file jamanetwopen-e2244772-s002.pdf]

**Data Sharing Statement**

**Wedderburn CJ, Ringshaw JE, Donald KA, et al. Association of maternal and child anemia with brain structure in early life in South Africa. *JAMA Netw Open*. 2022;5(12):e2244772. doi:10.1001/jamanetworkopen.2022.44772**

**Data**
**Data available:** No

**Additional Information**
**Explanation for why data not available:** The de-identified data that support the findings of this study are available from the authors upon reasonable request as per DCHS cohort guidelines.
